# Determining Optimum Conditions for Lipase-Catalyzed Synthesis of Triethanolamine (TEA)-Based Esterquat Cationic Surfactant by a Taguchi Robust Design Method

**DOI:** 10.3390/molecules16064672

**Published:** 2011-06-03

**Authors:** Hamid Reza Fard Masoumi, Anuar Kassim, Mahiran Basri, Dzulkifly Kuang Abdullah

**Affiliations:** Department of Chemistry, Faculty of Science, Universiti Putra Malaysia, 43400, Selangor, Malaysia

**Keywords:** Taguchi design, optimization, esterquat, cationic surfactant, biosynthesis

## Abstract

A Taguchi robust design method with an L_9_ orthogonal array was implemented to optimize experimental conditions for the biosynthesis of triethanolamine (TEA)-based esterquat cationic surfactants using an enzymatic reaction method. The esterification reaction conversion% was considered as the response. Enzyme amount, reaction time, reaction temperature and molar ratio of substrates, [oleic acid: triethanolamine (OA:TEA)] were chosen as main parameters. As a result of the Taguchi analysis in this study, the molar ratio of substrates was found to be the most influential parameter on the esterification reaction conversion%. The amount of enzyme in the reaction had also a significant effect on reaction conversion%.

## 1. Introduction

Triethanolamine (TEA)-based esterquats are potential high-value biodegradable products used as surfactants and softening agents. There have been an increasing number of researchers concerned with the study of biodegradable esterquat cationic surfactants since the beginning of the 1990s [1]. Due to the presence of the ester bond in the cationic surfactant, esterquat cationic surfactants can be degraded in the environment [2]. With the developing utilization of surfactants in both industry and daily life, esterquat cationic surfactants have attracted attention for their environmentally friendly, nontoxic and biodegradable properties. To date, esterquat cationic surfactants, aside from D1821, were mainly used as textile softening agents. The application of D1821 was limited by its pollution of the environment and high costs. Besides biodegradability additional advantages such as excellent softening properties, suitability for various fabrics and simple preparation procedures, have been discovered with the use of esterquat cationic surfactants as textile softening agents [1,3,4,5].

In the production of TEA-based esterquat cationic surfactants by the lipase-catalyzed synthesis method there are several factors that are important to obtain high conversion yields for the reaction. The conversions are affected by various parameters such as the enzyme amount, reaction time, reaction temperature and molar ratio of substrates (OA:TEA). The interrelationships between the above parameters are complex, and the analysis of this chemical reduction system to optimize the factors is a time and labor-consuming work. Hence, the analyses using conventional experimental methods are inefficient. 

Therefore the use of the Taguchi robust design method [6,7,8,9] was introduced in this paper. The Taguchi method is a combination of mathematical and statistical techniques used in an empirical study. It is economical for characterizing a complicated process. It requires fewer experiments to study all levels of all input parameters, and filters out some effects due to statistical variation. The Taguchi method can also determine the experimental condition having the least variability as the optimum conditions. Analysis of experimental data using the ANOVA (analysis of variance) gives the statistical relationships of the output. Aggarwal, Singh, Kumar, and Singh [10] reported that both the Taguchi and RSM (Response Surface Methodology) techniques predicted nearly similar results but the time required for conducting experiments using RSM technique was almost twice that needed for the Taguchi methodology. Benyounis and Olabi [11] reported that the Taguchi method was one of the powerful optimization techniques that could improve the product quality and reliability at low cost as compared to RSM and ANNs (Artificial Neural Networks).

## 2. Results and Discussion

Taguchi's approach to parameter design provides the design engineer with a systematic and efficient method for determining near optimum design parameters for performance and any response [12,13,14]. The objective is to select the best combination of control parameters so that the product or process is most robust with respect to noise factors. The Taguchi method utilizes orthogonal arrays from design of experiments theory to study a large number of variables with a small number of experiments. Using orthogonal arrays significantly reduces the number of experimental configurations to be studied. Furthermore, the conclusions drawn from small scale experiments are valid over the entire experimental region spanned by the control factors and their settings [12]. Orthogonal arrays are not unique to Taguchi, they were discovered considerably earlier [15]. However, Taguchi has simplified their use by providing tabulated sets of standard orthogonal arrays and corresponding linear graphs to fit specific projects [16,17].

### 2.1. Analysis of Experimental Data and Prediction of Performance

The influence of individual factors on TEA-based esterquat production and their performance at optimum condition using Taguchi approach were analyzed by software (Design Expert Version 6.0.6). The software is equipped to use from L–4 to L–64 arrays along with selection of 2–63 factors and their 2–5 levels. The automatic design option allows selecting the array used and assigning factors to the appropriate columns. Experimental data for lipase-catalyzed synthesis of TEA-based esterquat cationic surfactant with the predicted values are shown in Table 1. 

**Table 1:** Experimental data for lipase-catalyzed synthesis of TEA-based esterquat cationic surfactant with the predicted values.

Run No.	Enzyme Amount (w/w %)	Reaction Time (hour)	Reaction Temperature (°C)	Molar Ratio of Substrates (mole)	Conversion %	
					Actual	Predicted
1	0	−1	0	0	48.34	47.29
2	1	0	0	−1	46.27	45.37
3	−1	1	0	1	45.18	44.28
4	1	1	−1	0	42.32	43.01
5	−1	−1	−1	−1	38.98	39.51
6	1	−1	1	1	54.54	55.07
7	−1	0	1	0	45.95	46.64
8	0	0	−1	1	49.29	49.50
9	0	1	1	−1	39.94	40.15

**Figure 1:**
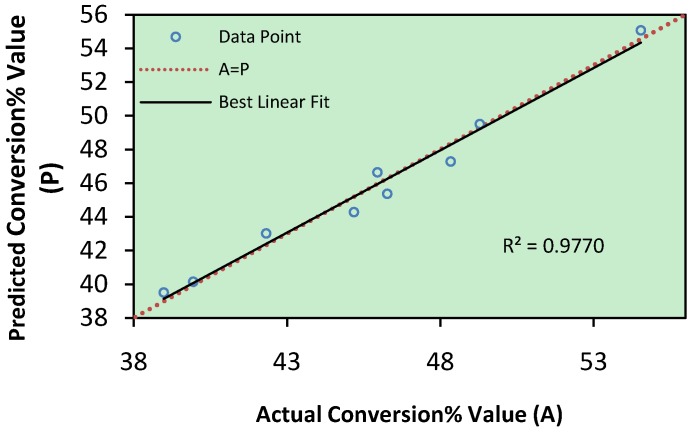
Scatter plot of predicted conversion% value *versus* actual conversion% value from Taguchi experimental design.

The predicted values were obtained from model fitting techniques were seen to be sufficiently correlated to observed values. Fitting of the data to various models (linear, quadratic, two factorial and cubic) and their subsequent ANOVA showed that reaction of esterification was most suitably described with a “linear” polynomial model. Figure 1 shows predicted value versus actual value of percentage conversion from Taguchi experimental design so that, the coefficient of determination (R^2^) of 0.9770 and the root mean squared error (RMSE) of 0.6932 indicated a good fit of the model.

### 2.2. Analysis of Variance (ANOVA)

The first step sequential F-tests were performed using analysis of variance (ANOVA), starting with a linear model and adding terms (linear). As shown in Table 2, under the "Source" column of the table, the line labeled "Linear" indicates the significance of adding mean terms. The F-value is calculated for each type of model, and the highest order model with significant terms normally would be chosen. Significance is judged by determining if the probability that the F-value calculated from the data exceeds a theoretical value. The probability decreases as the value of the F-value increases. If this probability is less than 0.05 the terms are significant and their inclusion improves the model [18]. In this study, the “linear” model is the highest order model with significant terms (P-value is less than 0.05); therefore, it would be the recommended model for this data. The 2FI (two factorial) model and higher were found to be aliased. Typically, the selected model will be the highest order polynomial where additional terms are significant and the model is not aliased [19]. 

**Table 2:** Statistical parameters for sequential models.

Source	Sum of Squares	Degree of Freedom	Mean Square	F Value	P Value	Remarks
Mean	18751.65	1	18751.65	-	-	-
Linear	173.61	4	43.40	11.74	0.0175	Suggested
2FI	12.22	3	4.07	1.59	0.5141	Aliased
Residual	2.56	0	-	-	-	-
Total	18940.05	9	2104.45	-	-	-

The regression method was used to fit the” linear” (modified) model to the experimental data and to identify the relevant model terms [20]. The response function to predict the percentage conversion of ester in the coded variables was as follows:

When a model has been selected, an analysis of variance (ANOVA) is calculated to assess how well the model represented the data [21]. The values of the coefficients and the analysis of variance (ANOVA) are presented in Table 3 and Table 4. 

**Table 3:** Analysis of Variance (ANOVA) and statistical parameters of TEA-based esterquat cationic surfactant reaction (Linear model).

Source	Sum of Squares	Degree of Freedom	Mean Square	F Value	P Value
Model	184.07	5	36.81	25.54	0.0116
Residual	4.32	3	1.44	-	-
Corrected Total	188.39	8	-	-	-
R-Squared		0.9770	Standard Deviation		1.20
Adjusted R^2^		0.9388	Coefficient of variation %		2.63
Adequate Precision		15.872	Predicted Residual Error of Sum of Squares (PRESS)		46.41

**Table 4:** Analysis of Variance (ANOVA) and Regression Coefficients of TEA-based esterquat cationic surfactant reaction (Linear model).

Source	Coefficient Estimate	Sum of Squares	Degree of Freedom	Mean Square	F Value	P Value
Intercept	47.17	-	-	-	-	-
X_1_	−2.40	34.66	1	34.66	24.04	0.0162
X_2_	1.64	16.14	1	16.14	11.19	0.0442
X_3_	2.17	28.25	1	28.25	19.60	0.0214
X_4_	3.97	94.57	1	94.57	65.60	0.0039
X_2_^2^	−2.29	10.46	1	10.46	7.25	0.0742

*Notes*: X_1_ = Enzyme Amount (w/w %), X_2_ = Reaction Time (hour), X_3_ = Reaction Temperature (°C), X_4_ = Molar Ratio of Substrates (mole).

The coefficients of the model were evaluated for significance with a Fisher’s F-test. The ANOVA indicates that the model F-value of 25.54 implied the model was significant. There was only 1.16% chance that the model F-value this large could occur due to noise. In general, the calculated F-value should be several times greater than the tabulated value for the model to be considered good [22]. The coefficient of determination (R^2^) of the model is obtained 0.9770, which indicates 97.70% of the variability in the response could be explained by the model. When R^2 ^approaches unity, the better empirical model fits the actual data [22]. Normally, a regression model, having an R^2^-value higher than 0.9 is considered as model having a very high correlation [23]. The present R^2^-value, therefore, reflected a very good fit between experimental and predicted values. The adjusted determination coefficient (Adjusted R^2^ = 0.9388) was also satisfactory, confirming the significance of the model. The Coefficient of Variation (CV) as the ratio of the standard error of estimate to the mean value of the observed response (as a percentage) is a measure of reproducibility of the model and as a general rule a model can be considered reasonably reproducible if its CV is not greater than 10% [24]. A lower value of coefficient variation (CV = 2.63%) clearly showed a high degree of precision and a good deal of reliability of the experimental values [25]. The model also showed adequate precision by the measured the signal to noise ratio. The ratio greater than 4 is desirable. Thus, a ratio of 15.87 indicated an adequate signal. This model can be used to navigate the design space.

The P-values are used as a tool to check significance of each variable, which also indicate the interaction strength between each independent variable [26]. The smaller P-values show the bigger the significance of the corresponding variable [18]. P-values in this study less than 0.05 indicate model terms are significant. From the results obtained in Table 4, all the linear coefficients, and the quadratic term were significant model terms (P-value less than 0.05). The final model to predict the percentage of conversion of TEA-based esterquat cationic surfactant reaction catalyzed by Novozyme 435 is shown in Equation (1).

Negative values of coefficient estimates denote negative influence of parameters on the reaction. It was observed that all the linear coefficients of the model gave positive effect except coefficient estimate for enzyme amount (X_1_) in the model of percentage conversion. This may be due to the percentage of conversion was negatively affected by the presence of higher amount of enzyme as the ratio of ester amount/ initial amount of enzyme is lower at higher enzyme amount compared to lower enzyme amount. Besides, it was observed that has significant effect to the reaction. Indeed, despite the negative value, amount of enzyme has one of the biggest effects to response after the effect of molar ratio of substrates.

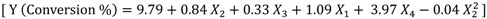
(1)
where *Y* = Percentage of Conversion, *X_1_* = Amount of Enzyme, *X_2_* = Reaction Time, *X_3_* = Reaction Temperature and *X_4_* = Substrate Molar Ratio (OA: TEA)

### 2.3. Optimization of Reaction and Model Validation

Three solutions with different desirability values were used to predict the optimal conditions for Novozyme-catalyzed production of TEA-based esterquat cationic surfactants and they are presented in Table 5. Experiments were then carried out under the recommended conditions and the resulting responses were compared to the predicted values. The largest reaction conversion% (49.94%) was obtained in experiment number 3 compared to the other two experiments. The optimum reaction parameters were: enzyme loading of 5.50 wt % of oleic acid, amount of oleic acid of 17.70 mmol, amount of triethanolamine of 8.85 mmol (molar ratio of substrates 1:2), reaction time of 14.44 hours and reaction temperature of 61 °C. The relative deviation of 3.14% is also obtained from the Taguchi experimental design. Comparison of predicted and actual values revealed good correspondence between them, implying that the empirical model derived from Taguchi experimental design can be used to adequately describe the relationship between the factors and the response in Novozyme- catalyzed synthesis of TEA-based esterquat cationic surfactant. 

**Table 5:** Optimum conditions derived by Taguchi design for TEA-based esterquat cationic surfactant synthesis.

Exp.	Optimal Conditions				Conversion %		
	X_1_	X_2_	X_3_	X_4_	Actual	Predicted	Relative Deviation
1	5.50	14.06	61.00	2.00	47.34	48.49	2.37
2	5.50	14.00	60.83	2.00	46.27	48.44	4.48
3	5.50	14.44	61.00	2.00	49.94	48.42	3.14

*Note*s: X_1_ = Enzyme Amount (w/w %), X_2_ = Reaction Time (hour), X_3_ = Reaction Temperature (°C), X_4_ = Molar Ratio of Substrates (mole).

The lower amount of Novozyme 435 is required to produce the respective amount of product in the aforementioned experiment. From the process point of view, it would be desirable to use lowest enzyme amount to achieve maximum conversion of substrate [27]. This is because Novozyme 435 is more expensive that the other substrates, thus high reaction conversion obtained by low amount of enzyme. Moreover, shorter reaction time and lower reaction temperature were considered in optimization process because longer reaction time and higher reaction temperature lead to enzyme denaturation and both could resulted in lower reaction conversion especially in longer reaction time.

## 3. Experimental

### 3.1. Materials

Novozyme 435, Candida *antarctica* lipase B immobilized on a macroporous acrylic resin (10,000 propyl laurate units per gram), was purchased from Novo Nordisk A/S (Bagsværd, Denmark). The enzyme is a granular product with a particle size of 0.2– 0.6 mm. The bulk density of Novozyme 435 is 350–450 kg/m^3^. *n*-Hexane obtained from J.T. Baker (USA) was used as the organic solvent. Oleic acid and triethanolamine were purchased from Merck (Germany). All other chemicals used in this study were of analytical reagent grade.

### 3.2. Method

Enzymatic esterification and analysis of samples

The reactions were performed in 50 mL flasks and specified volumes of hexane were added as solvent. Different molar ratios of oleic acid and triethanolamine were mixed and different amounts of lipase were subsequently added. The reaction was performed under reflux system on magnetic stirring at different temperatures and for different time periods, as shown in Table 6. 

**Table 6:** Variables and their levels employed in the Taguchi robust design method.

Variable		Units	Coded Level of Variable		
			−1	0	1
X_1_	Enzyme Amount	%w/w	3	5	7
X_2_	Reaction Time	Hour	8	16	24
X_3_	Reaction Temperature	°C	55	60	65
X_4_	Molar ratio of substrates	OA:TEA (mole:mole)	1:1	2:1	3:1

At the end of the experiment periods, the reaction was terminated by dilution with ethanol-acetone (30 mL, 50:50, v/v). The enzyme particles were then separated by filtration and the remaining free acid in the reaction mixture was determined by titration with 0.1M NaOH in the presence of phenolphthalein and a pH meter. The moles of acid reacted were calculated from the values obtained for the blank (without enzyme) and test samples. The ester formed was expressed as equivalent to conversion of the acid. The accuracy of the method to follow ester formation was confirmed using thin layer chromatography.

## 4. Conclusions

A Taguchi robust design method with an L_9_ orthogonal array was implemented to optimize the experimental conditions for the synthesis of TEA-based esterquat cationic surfactants using a lipase catalyzed synthesis method. Enzyme amount, reaction time, reaction temperature and molar ratio of substrates (OA:TEA) were chosen as main parameters. As a result, molar ratio of substrates (OA:TEA) and enzyme amount were the main parameters having a significant effect on percentage conversion of the reaction. A percentage conversion of 49.94% was obtained using a low amount of enzyme, which matched well with the predicted value of 48.42%. Therefore, this study serves as another example for the application of the Taguchi methodology for improvement of a biotechnological process. 
